# Associations of Electromechanical Activation Time With Cardiac Structure and Function and Clinical Outcomes

**DOI:** 10.1016/j.jacadv.2025.102371

**Published:** 2025-11-27

**Authors:** Jake Munch, Colby R. Ayers, Justin L. Grodin, Jennifer T. Thibodeau, Nicholas S. Hendren, Faris G. Araj, Elizabeth A. Hardin, James A. de Lemos, Amil M. Shah, Ambarish Pandey, Mark H. Drazner

**Affiliations:** a University of Texas Southwestern Medical School, University of Texas Southwestern Medical Center, Dallas, Texas, USA; b Division of Cardiology, Department of Internal Medicine, University of Texas Southwestern Medical Center, Dallas, Texas, USA

**Keywords:** heart failure, left ventricular ejection fraction, left ventricular hypertrophy, natriuretic peptide, prognosis, screening

## Abstract

**Background:**

The electromechanical activation time (EMAT) measured by acoustic cardiography is the time between QRS onset and the first heart sound. Increased EMAT is associated with a reduced left ventricular ejection fraction (LVEF).

**Objectives:**

The objective of the study was to determine whether EMAT has utility as a cardiovascular (CV) screening test in the community.

**Methods:**

Participants (n = 3,401) in Dallas Heart Study-2 had acoustic cardiography, cardiac magnetic resonance imaging, and blood sampling at baseline. Clinical outcomes, including a composite of CV death/heart failure (HF) hospitalization or major adverse clinical events (CV death, HF hospitalization, nonfatal myocardial infarction, stroke, and atrial fibrillation), were ascertained after a median follow-up of 12.4 years. Multivariable models evaluated whether EMAT was associated with cardiac structure and function and clinical outcomes. Discrimination for reduced LVEF (<55%) was assessed by C-statistics.

**Results:**

Higher continuous EMAT was independently associated with a lower LVEF (*P* < 0.001) and greater left ventricular end-diastolic volume (*P* = 0.04). However, an increased EMAT had poor discrimination for reduced LVEF (C-statistic = 0.52), was inferior to N-terminal pro-B-type natriuretic peptide (NT-proBNP) (C = 0.59, *P* < 0.01 vs EMAT), and did not improve discrimination beyond NT-proBNP alone (*P* = 0.90). Baseline EMAT was associated with CV death/HF hospitalization (HR: 1.3; 95% CI: 1.2-1.4; *P* < 0.001) and with major adverse clinical events (HR: 1.2; 95% CI: 1.1-1.4; *P* < 0.001) in models adjusted for age, race, and sex, but these associations did not persist after additional adjustment for NT-proBNP (*P* > 0.10 for both).

**Conclusions:**

In a community cohort, an increased EMAT was associated with a lower LVEF and adverse prognosis but had poor discriminatory capacity for the former and was not independent of NT-proBNP for the latter.

Remote monitoring devices offer promise to improve clinical outcomes in patients with heart failure (HF). Implantable pulmonary artery pressure monitors and lead-based devices incorporated into pacemakers or implantable cardiac defibrillators have already entered the clinical arena. Acoustic cardiography is another remote patient monitoring technology being tested for this purpose with 1 device, Audicor RPM (Inovise Medical, Oregon), a hand-held device usable in the home, receiving Food and Drug Administration Breakthrough Device designation in 2021.^1^^,^^2^

Acoustic cardiography can record cardiac heart sounds from cardiac sensors on the chest wall, building on the historical modality of phonocardiography, while simultaneously acquiring a standard electrocardiogram (ECG). Parameters measured by acoustic cardiography have been termed “cardiac acoustic biomarkers”^3^ and include a third heart sound (S_3_ gallop), an accepted risk marker in patients with chronic HF,^4^ and a variety of intervals determined by the time between electrocardiographic complexes (eg, QRS) and/or the heart sounds which signify specific events in the cardiac cycle.

One such cardiac acoustic biomarker is the electromechanical activation time (EMAT), calculated as the time from the QRS onset to the first heart sound (S_1_). EMAT assesses the mechanical function of the left ventricle^5^^,^^6^ by measuring the time from onset of electrical depolarization to that when left ventricular (LV) intracavitary pressure increases adequately to close the mitral valve and result in S_1_. EMAT is often standardized to the percent of the cardiac cycle, expressed as the RR interval in msec, and herein termed EMATc.

Higher EMAT or EMATc have been associated with lower LV ejection fraction (LVEF) in many^6^, ^7^, ^8^, ^9^ but not all^3^ studies. These parameters have also been associated with a lower LV dP/dt^5^ and a higher E/e’ ratio.^10^ EMATc demonstrated diagnostic utility in discriminating patients with HF with preserved ejection fraction from those with hypertension (HTN).^11^ In patients with suspected HF and a B-type natriuretic peptide (BNP) between 100 to 500 pg/mL, EMATc increased the sensitivity and specificity for detection of systolic or diastolic dysfunction.^12^ In patients with HF, EMAT was associated with adverse clinical outcomes.^13^, ^14^, ^15^

Although well studied in patients with suspected or known cardiovascular conditions,^9^^,^^10^^,^^12^^,^^14^, ^15^, ^16^ to our knowledge, the utility of acoustic cardiography has not previously been evaluated in a well-characterized community cohort. We performed acoustic cardiography in the phase 2 visit of the Dallas Heart Study (DHS), a study of over 3,000 participants, who also had deep phenotyping including cardiac magnetic resonance imaging (CMRI), assessments of body composition, assessments of coronary artery calcium, and measurement of N-terminal pro-BNP (NT-proBNP) and high-sensitivity troponin T (Hs-cTnT) levels. We hypothesized that acoustic cardiography parameters, and specifically EMAT, would be associated with cardiac structure and function and an increased risk of subsequent adverse clinical outcomes. We were also interested in testing whether such associations, if present, were independent of other potential measures of risk such as natriuretic peptide levels. If such associations were present, they would provide a foundation to consider acoustic cardiography as a screening tool of risk in the community.

## Methods

### Cohort

The DHS-1 was a probability-based, multiethnic cohort study of Dallas county residents aged 18 to 65 years that was designed to intentionally oversample Black individuals in an effort to enroll an equal percentage of Black and non-Black study participants between 2000 and 2002.^17^ From 2007 to 2009, DHS-1 participants (n = 2,485) and their spouses or other unrelated individuals (n = 916) participated in the phase 2 visit of the DHS-2 (NCT00344903) which included blood pressure measurements, collection of blood and urine samples, CMRI, coronary artery calcium scanning, and acoustic cardiography. Not all 3,401 DHS-2 participants completed all the previously mentioned testing. Participants provided written informed consent. The UT Southwestern Institutional Review Board approved the DHS-2 study.

### Acoustic cardiography

Acoustic cardiography was performed using the Audicor system in 3,302 DHS-2 participants. Two Audicor acoustic sensors were placed on each participant’s chest wall at the position of V_3_ and V_4_ with the remaining 10 leads in the standard ECG configuration. The Audicor system measures EMAT. Three separate 10-second readings were performed consecutively. We report the average of these 3 readings, both for EMAT and for heart rate. EMATc was calculated as: EMATc = EMAT × (ventricular rate/60,000). To define the threshold for an abnormal EMAT and EMATc in the current study, we assessed its value at the 95th percentile in the overall cohort as well as in a healthy normal subpopulation (n = 165). The latter was defined by the following criteria: no self-reported history of HF, myocardial infarction, valvular heart disease, or congenital heart disease; body mass index (BMI) <35 kg/m^2^; no history of HTN of diabetes mellitus; coronary artery calcium score of 0; high-sensitivity (Hs)-troponin I levels of 0 ng/L;^18^ and a LVEF between 55% and 77%. The 95th percentile EMAT values in the overall cohort and the healthy subpopulation were within 5% of each other; we averaged them and rounded to whole numbers resulting in an elevated EMAT being defined as ≥110 msec and EMATc as ≥14%.

### Cardiac magnetic resonance imaging

CMRI was performed as described previously.^19^ We assessed LVEF, LV mass, LV end-diastolic volume (LVEDV), LV concentricity (LV mass/LVEDV), LV concentricy^0.67^ (LV mass/LVEDV^0.67^), and average LV wall thickness. LVEF was defined as reduced if it was <55% (N = 65, 3.2% of cohort).

### N-terminal pro-B-type natriuretic peptide

NT-proBNP was measured as previously described.^19^, ^20^, ^21^ For this study, we defined gender-specific thresholds for elevated NT-proBNP levels based on the 97.5th percentile values reported in those aged 50 to 59 years of age in a large general population cohort: ≥200 pg/mL (men) and ≥300 pg/mL (women).^22^

### High-sensitivity troponin T

Hs-cTnT was measured using a highly sensitive assay on an automated platform (Elecsys Troponin T hs STAT; Roche Diagnostics) as previously described,^23^ with limit of blank of 3 ng/L, a limit of detection of 5 ng/L, and a reported 99th percentile value in apparently healthy individuals of 14 ng/L.

### Coronary artery calcium

Coronary artery calcium (CAC) was measured via multidetector computed tomography as described previously.^24^

### Clinical outcomes

Clinical outcomes were ascertained in DHS-2 participants via direct annual contact of participants as well as querying the National Death Index and the Dallas Fort Worth Hospital Council as described previously.^21^^,^^25^ Fatal and nonfatal events were adjudicated by a panel of cardiology experts. The median follow-up was 12.4 years. In this study, clinical outcomes were: 1) a composite of cardiovascular death/HF hospitalization; and 2) major adverse cardiac events (MACE) defined as cardiovascular death, nonfatal myocardial infarction, stroke, incident HF, and atrial fibrillation.

### Statistical analysis

All statistical analyses were conducted using SAS (version 9.4) software. Continuous variables are expressed as mean ± SD or median [25th, 75th percentile], as appropriate. Trends across increasing EMAT quartile were assessed by the Jonckheere-Terpstra test for continuous variables and by the Cochran-Armitage test for categorical variables. Multivariable-adjusted linear regression models were constructed to evaluate the association between mean EMAT and EMATc with cardiac structure and function. Standardized β-estimates are presented for continuous variables such that the mean is 0 and the SD is 1 for each variable. The presented β-estimates thus represent the effect of 1 SD increment. Covariates adjusted for included age, race, sex, QRS duration, ECG left ventricular hypertrophy, diabetes, systolic blood pressure, hypertensive status, hemoglobin A1c, CAC, estimated glomerular filtration rate (eGFR), height, fat mass, lean mass, history of myocardial infarction, NT-proBNP, high-sensitivity-troponin, and, for EMAT models, heart rate. High-sensitivity troponin was entered as a log transformed continuous variable, with undetectable values (<3) set at 1.5 by convention as previously.^23^ We also present the standardized β-estimates for heart rate given qualitatively differential associations of cardiac structure with EMAT and EMATc. Kaplan-Meier curves for the survival outcomes of MACE and CV death and HF hospitalization were constructed by quartiles of EMAT, EMATc, and heart rate. A series of multivariable Cox regression analyses were also performed to determine the association of the mean EMAT alone, EMAT with heart rate, heart rate alone, mean EMATc, or log NT-proBNP with either CV death/HF hospitalization or overall MACE. Hazard ratios (HRs) are based on are based on 1 SD change of the exposure variable. These models included age, race, sex, log NT-proBNP level (in the EMAT, EMATc, or heart rate models), Hs-troponin, QRS duration, eGFR, diabetes, HTN status, CAC score, and BMI. The Cox proportional hazards assumptions were satisfied by Schoenfeld residuals. C-statistics for EMAT, EMATc, and NT-proBNP were determined by receiver-operating characteristic (ROC) analysis via logistic regression with low LVEF as the outcome. These C-statistics were compared with the nonparametric test of DeLong, DeLong, and Clarke-Pearson.

## Results

**Figure 1:**
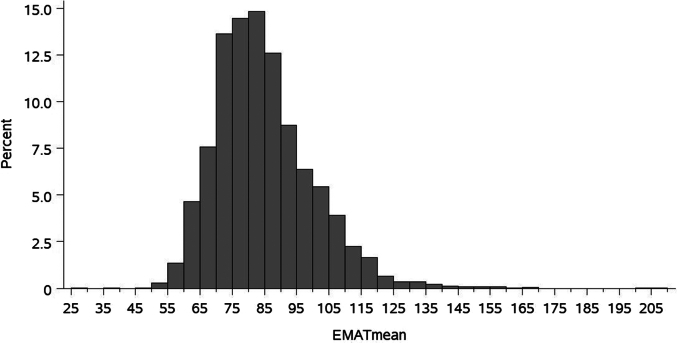
**Histogram of Electromechanical Activation Time** The distribution of EMAT (msec) in the cohort is shown. The mean ± SD EMAT was 84.6 ± 15.5. EMAT = electromechanical activation time.

Among the 3,401 original study participants, 3,302 had acoustic cardiography performed. Of these, 198 (6%) had an abnormal EMAT (>110 msec) and 142 (4.3%) had an abnormal EMATc (>14%). EMAT quartiles were defined as Q1 (28-73.7), Q2 (74-82.3), Q3 (82.5-92.7), and Q4 (93-208 ms). The distribution of EMAT is shown in the overall cohort (Figure 1). Quartiles heart rates were defined (35-60, 60.3-66.7, 67-74.5, and 74.7-117 beats per minute).

Baseline characteristics by EMAT quartiles are shown in Table 1. Increasing EMAT quartile was associated with older age, female sex, higher systolic blood pressure and BMI, lower heart rate, prevalent HF, diabetes, HTN, antihypertensive use, increased fat mass, decreased lean mass, and increased QRS duration. Higher EMAT also was associated with the increasing NT-proBNP, Hs-cTnT, hemoglobin A1c, QRS duration, and with the lower eGFR. EMAT was weakly correlated with NT-proBNP levels (r = 0.018; *P* < 0.001).

**Table 1:** Baseline Characteristics by Quartile EMAT

	Cohort (N = 3,301)	EMAT (ms) Quartile				
		Q1 (28-73.7) (n = 817)	Q2 (74-82.3) (n = 845)	Q3 (82.5-92.7) (n = 819)	Q4 (93-208) (n = 820)	*P* Value^a^
EMAT, ms	85 ± 15	68 ± 6	78 ± 3	87 ± 3	105 ± 12	Definitional
EMATc, %	9.6 ± 3	7.8 ± 2	8.9 ± 2	9.8 ± 2	11.8 ± 3	Definitional
Age, years	50 ± 11	48 ± 11	50 ± 11	50 ± 11	52 ± 11	<0.001
Race						
Black	51%	48%	49%	54%	52%	0.03
White	32%	33%	33%	29%	34%	1.0
Female	59%	53%	57%	61%	66%	<0.001
Total fat mass, kg	32.6 ± 12.5	31.9 ± 11.7	32.2 ± 13.3	32.2 ± 12.5	33.8 ± 12.5	0.001
Total lean mass, kg	51.6 ± 11.3	52.1 ± 11.2	52.3 ± 11.2	51.3 ± 11.7	50.5 ± 10.9	<0.001
BMI, kg/m^2^	31 ± 7.5	31 ± 7.5	31 ± 7.5	31 ± 7.9	32 ± 7.8	0.03
Systolic blood pressure (mm Hg)	133 ± 20	131 ± 18	132 ± 19	133 ± 20	134 ± 22	0.03
Hypertension	51%	44%	52%	52%	55%	<0.001
Antihypertensive medication	35%	27%	35%	37%	41%	<0.001
H/o HF	2.5%	0.9%	2%	2%	5%	<0.001
NT-proBNP (pg/mL)	43 (25, 81)	35 (21, 61)	42 (24, 77)	49 (26, 89)	53 (29, 108)	<0.001
Hs-cTnT (pg/mL)	5.3 (1.5, 8.9)	4.7 (1.5, 8)	5 (1.5,8.2)	5.6 (3, 9.1)	6 (3, 10)	<0.001
eGFR (ml/min/1.73 m^2^)	59 ± 5	60 ± 3.4	59 ± 4	59 ± 6	58 ± 6	<0.001
Diabetes	16%	13%	16%	15%	21%	<0.001
HbA1c (%)	5.8 ± 1.1	5.7 ± 1.1	5.7 ± 1.1	5.7 ± 1.1	5.9 ± 1.3	0.03
CAC (Agatston units)	0 (0, 22)	0 (0, 12)	0 (0, 22)	0 (0, 22)	0 (0, 39)	0.03
Heart rate, beats/min	68 ± 13	69 ± 13	68 ± 13	67 ± 11	67 ± 15	<0.001
QRS duration, ms	93 ± 13	90 ± 11	93 ± 12	93 ± 12	98 ± 16	<0.001
PR interval, ms	158 ± 31	160 ± 25	156 ± 28	155 ± 27	159 ± 40	0.8

BMI = body mass index; CAC = coronary artery calcium; CHF = congestive heart failure; eGFR = estimated glomerular filtration rate; EMAT = electromechanical activation time; EMATc = electromechanical activation time standardized to percent of cardiac cycle; HbA1c = hemoglobin A1c; Hs-cTnT = high-sensitivity troponin T; NT-proBNP = N-terminal pro-B-type natriuretic peptide.

Values are median (Q1, Q3), mean ± SD, or %, as appropriate.

a For comparison across quartiles.

We next assessed the association of EMAT with cardiac structure and function (Table 2). Increasing quartile EMAT was associated with lower LVEF and higher LV mass/LVEDV. In contrast, there was no association of quartile EMAT with LV mass, LVEDV, LV wall thickness, LV mass/EDV^0.67^, LV mass/body surface area, LV mass/height, and LV mass/height^0.67^. In multivariable models, EMAT was associated with lower LVEF and increased LVEDV and LV mass in the fully adjusted model (Table 3). There was no association of EMAT with LV wall thickness, LV mass/EDV, or LV mass/EDV^0.67^ in fully adjusted multivariable models (Table 3). An abnormal EMAT had poor discrimination of reduced LVEF by ROC analysis in the overall cohort (C-statistic = 0.52), among those with diabetes (C-statistic = 0.60), HTN (C-statistic = 0.54), or those older than 50 years of age (C-statistic = 0.52). The C-statistic for elevated NT-proBNP (gender-specific cutpoints) for detection of low LVEF was higher than that for abnormal EMAT in the overall cohort (C-statistic = 0.59, *P* = 0.01). Adding abnormal EMAT to elevated NT-proBNP in the ROC model did not increase the C-statistic for the detection of low LVEF (*P* = 0.9) vs elevated NT-proBNP alone. Continuous EMAT was weakly correlated with LVEF (r = −0.08; *P* < 0.001) despite their significant association present in multivariable models (Table 3).

**Table 2:** Association of Quartile EMAT With Cardiac Structure and Function

	Cohort (N = 2,023)	EMAT Quartile				
		Q1 (n = 523)	Q2 (n = 522)	Q3 (n = 510)	Q4 (n = 468)	*P* Value^a^
LV mass, g	128 ± 38	127 ± 33	129 ± 35	128 ± 39	130 ± 43	0.6
LVEDV mL	119 ± 28	116 ± 25	119 ± 26	119 ± 30	121 ± 32	0.2
LVWT mm	7.5 ± 1.5	7.5 ± 1.4	7.5 ± 1.5	7.5 ± 1.6	7.6 ± 1.7	0.5
LV mass/LVEDV (g/mL)	1.09 ± 0.25	1.10 ± 0.24	1.09 ± 0.23	1.08 ± 0.25	1.08 ± 0.29	0.04
LV mass/EDV^0.67^ (g/mL^0.67^)	5.2 ± 1.2	5.3 ± 1.1	5.3 ± 1.1	5.2 ± 1.2	5.2 ± 1.3	0.1
LV mass/BSA (g/m^2^)	65 ± 16	64 ± 15	66 ± 15	65 ± 16	66 ± 19	0.7
LVEDV/BSA (mL/m^2^)	61 ± 12	59 ± 11	61 ± 11	61 ± 13	62 ± 14	0.09
LV mass/height (g/m)	76 ± 20	75 ± 17	76 ± 19	76 ± 21	77 ± 23	0.9
LV mass/height^2.7^ (g/m^2.7^)	31 ± 8	31 ± 7	31 ± 7	31 ± 8	32 ± 9	0.5
LVEF (%)	69 ± 7	70 ± 6	69 ± 7	69 ± 7	68 ± 7	<0.001
Reduced (<55%) LVEF	2.5%	0.8%	2.5%	2.8%	4.1%	0.01

BSA = body surface area; LV = left ventricular; LVEF = left ventricular ejection fraction; LVEDV = left ventricular end-diastolic volume; LVWT = left ventricular wall thickness.

Values are mean ± SD or %.

a For comparison across quartiles.

**Table 3:** Association of EMAT With Cardiac Structure and Function in Multivariable Analysis

	LVEF		LV Mass		LVEDV		LV Mass/EDV		LV Mass/LVEDV^0.67^		LVWT	
	β	*P* Value	β	*P* Value	β	*P* Value	β	*P* Value	β	*P* Value	β	*P* Value
Model 1	−0.16 (−0.21 to −0.12)	<0.001	0.12 (0.08-0.16)	<0.001	0.14 (0.10-0.18)	<0.001	0.006 (−0.04 to 0.05)	0.80	0.05 (0.01-0.09)	0.009	0.08 (0.04-0.12)	<0.001
Model 2	−0.14 (−0.18 to −0.09)	<0.001	0.07 (0.03-0.10)	<0.001	0.08 (0.005-0.13)	<0.001	0.004 (−0.05 to 0.03)	0.90	0.03 (−0.02 to 0.06)	0.16	0.05 (0 to 0.08)	0.02
Model 3	−0.15 (−0.21 to −0.10)	<0.001	0.07 (0.03-0.11)	<0.001	0.07 (0.03-0.12)	0.001	0.01 (−0.04 to 0.06)	0.70	0.03 (−0.01 to 0.08)	0.13	0.05 (0.002-0.09)	0.03
Model 4	−0.13 (−0.19 to −0.06)	<0.001	0.07 (0.02-0.11)	0.003	0.05 (0.003-0.10)	0.04	0.02 (−0.04 to 0.08)	0.50	0.04 (−0.02 to 0.10)	0.16	0.04 (−0.01 to 0.09)	0.14
Model 5	−0.13 (−0.20 to −0.06)	<0.001	0.06 (0.02-0.11)	0.005	0.05 (0.003-0.11)	0.04	0.02 (−0.05 to 0.08)	0.60	0.04 (−0.02 to 0.09)	0.18	0.03 (−0.02 to 0.09)	0.16

LVH = left ventricular hypertrophy; other abbreviations as in Tables 1 and 2.

Standardized β estimates (95% CI) for the association of EMAT with the various parameters of cardiac structure and function are reported. The SD of EMAT is 15.5.

Model 1: Controlled for age, sex, race.

Model 2: Model 1 + QRS duration, ECG LVH, heart rate.

Model 3: Model 2 + diabetes, systolic blood pressure, hypertensive status, hemoglobin A1c, CAC, eGFR, height, fat mass, lean mass, history of MI.

Model 4: Model 3 + NT-proBNP.

Model 5: Model 4 + Hs-troponin.

Similar multivariable analyses testing the association of EMATc with cardiac structure and function were performed (Table 4). As with EMAT, EMATc was associated with lower LVEF and higher LV mass. However, in contrast to EMAT, EMATc was inversely associated with LVEDV. Furthermore, EMATc was significantly associated with markers of concentric hypertrophy (increased LV wall thickness, LV mass/EDV, or LV mass/EDV^0.67^) in fully adjusted models. Abnormal EMATc, as with EMAT, had poor discrimination of reduced LVEF in the overall cohort and designated subgroups in ROC analysis with C-statistics ranging from 0.53 to 0.65. As with EMAT, EMATc was very weakly correlated with LVEF (r = −0.06; *P* = 0.01) despite their significant association present in multivariable models (Table 4).

**Table 4:** Association of EMATc With Cardiac Structure and Function in Multivariable Analysis

	LVEF		LV Mass		LVEDV		LV Mass/EDV		LV Mass/LVEDV^0.67^		LVWT	
	β	*P* Value	β	*P* Value	β	*P* Value	β	*P* Value	β	*P* Value	β	*P* Value
Model 1	−0.16 (−0.21 to −0.10)	<0.001	0.07 (0.04-0.10)	<0.001	−0.06 (−0.1 to −0.02)	0.004	0.18 (0.13-0.22)	<0.001	0.15 (0.11-0.19)	<0.001	0.15 (0.1-0.19)	<0.001
Model 2	−0.14 (−0.19 to −0.10)	<0.001	0.05 (0.01-0.09)	0.013	−0.09 (−0.1 to −0.05)	<0.001	0.17 (0.12-0.21)	<0.001	0.13 (0.09-0.18)	<0.001	0.13 (0.08-0.17)	<0.001
Model 3	−0.2 (−0.3 to −0.1)	<0.001	0.04 (0.005-0.08)	0.03	−0.08 (−0.1 to −0.03)	0.002	0.14 (0.09-0.2)	<0.001	0.12 (0.07-0.16)	<0.001	0.11 (0.06-0.15)	<0.001
Model 4	−0.15 (−0.20 to −0.09)	<0.001	0.06 (0.01-0.1)	0.02	−0.07 (−0.1 to −0.02)	0.008	0.16 (0.09-0.22)	<0.001	0.13 (0.07-0.19)	<0.001	0.11 (0.05-0.16)	<0.001
Model 5	−0.16 (−0.20 to −0.09)	<0.001	0.05 (0.006-0.1)	0.03	−0.07 (−0.1 to −0.02)	0.008	0.15 (0.09-0.22)	<0.001	0.12 (0.07-0.18)	<0.001	0.11 (0.05-0.16)	<0.001

Abbreviations as in Tables 1 and 2.

Standardized β estimates (95% CI) for the association of EMATc with the various parameters of cardiac structure and function are reported. The SD of EMATc is 0.096.

Model 1: Controlled for age, sex, race.

Model 2: Model 1 + QRS duration, ECG LVH.

Model 3: Model 2 + diabetes, systolic blood pressure, hypertensive status, hemoglobin A1c, CAC, eGFR, height, fat mass, lean mass, history of MI.

Model 4: Model 3 + NT-proBNP.

Model 5: Model 4 + Hs-troponin.

To determine the basis of the qualitatively different patterns of associations of EMAT and EMATc with cardiac structure in multivariable models, that is, EMAT was associated with larger LVEDV and EMATc with smaller LVEDV; and EMATc, but not EMAT, was associated with markers of concentric hypertrophy, we tested the association of heart rate with cardiac structure (Table 5). In fully adjusted models, the heart rate was inversely associated with LVEDV and positively associated with the 3 markers of concentric hypertrophy (*P* < 0.001 for each).

**Table 5:** Association of Heart Rate With Cardiac Structure and Function in Multivariable Analysis

	LVEF		LV Mass		LVEDV		LV Mass/EDV		LV Mass/LVEDV^0.67^		LVWT	
	β	*P* Value	β	*P* Value	β	*P* Value	β	*P* Value	β	*P* Value	β	*P* Value
Model 1	−0.05 (−0.10, −0.01)	0.02	0.01 (−0.04, 0.03)	0.50	−0.20 (−0.23, −0.15)	<0.001	0.20 (0.17, 0.26)	<0.001	0.14 (0.11, 0.19)	<0.001	0.10 (0.08, 0.16)	<0.001
Model 2	−0.12 (−0.18, −0.05)	<0.001	−0.03 (−0.07, 0.02)	0.30	−0.20 (−0.30, −0.18)	<0.001	0.20 (0.16, 0.28)	<0.001	0.14 (0.09, 0.19)	<0.001	0.10 (0.05, 0.16)	<0.001
Model 3	−0.10 (−0.17, −0.02)	0.01	−0.004 (−0.05, 0.05)	0.90	−0.19 (−0.25, −0.10)	<0.001	0.20 (0.15, 0.29)	<0.001	0.15 (0.08, 0.20)	<0.001	0.10 (0.06, 0.18)	<0.001

Abbreviations as in Table 2.

Standardized β estimates (95% CI) for the association of heart rate (HR) with the various parameters of cardiac structure and function are reported. The SD of HR was 10.6.

Model 1: Controlled for age, sex, race, EMAT.

Model 2: Model 1 + QRS duration, ECG LVH, diabetes, systolic blood pressure, hypertensive status, hemoglobin A1c, CAC, eGFR, height, fat mass, lean mass, history of MI.

Model 3: Model 2 + NT-proBNP, Hs-troponin.

**Figure 2:**
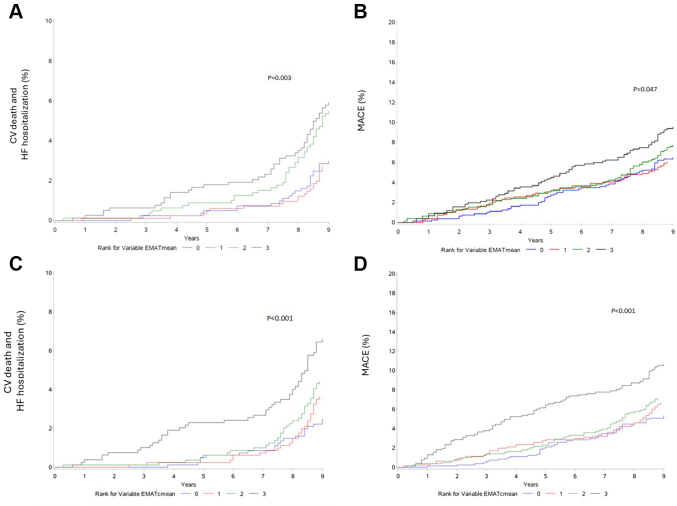
Association of EMAT and EMATc With Clinical Outcomes Increasing quartile of EMAT was associated with the composite endpoint of cardiovascular death or heart failure hospitalization (panel A) and major adverse cardiac events (CV death, nonfatal myocardial infarction, stroke, incident heart failure, and atrial fibrillation) (panel B). Likewise, increasing quartile of EMATc was associated with these outcomes (panels C and D, respectively). CV = cardiovascular; EMATc = electromechanical activation time standardized to percent of cardiac cycle; HF = heart failure; MACE = major adverse cardiovascular events; other abbreviations as in Figure 1.

We next tested the association of EMAT, EMATc, and heart rate with clinical outcomes. Over a median follow-up of 12.4 years, 137 participants experienced CV death or HF hospitalization and 233 participants experienced MACE. Quartiles of EMAT (Figure 2), EMATc (Figure 2), and heart rate (Supplemental Figure 1), were associated with CV death/HF hospitalization and MACE. In multivariable models, continuous EMAT was associated with both outcomes in models adjusted for age, race, and sex (Table 6). These associations persisted when participants with a history of HF at baseline were excluded (data not shown). However, in the overall cohort, continuous EMAT was no longer associated with clinical outcome once NT-proBNP was entered as a covariate (model 1 in Table 6). In contrast, continuous EMATc remained associated with both outcomes in models adjusting for age, race, sex, NT-proBNP, Hs-troponin, QRS duration, and eGFR (model 2 in Table 6). In comparable multivariable models with the same covariates as model 2, an elevated (dichotomous) EMATc was associated with CV death/HF with HR 2.4 (95% CI: 1.1-5.3; *P* = 0.03) and MACE with HR 2.9 (95% CI: 1.4-6.0; *P* = 0.004). Elevated (dichotomous) EMATc remained associated with clinical outcome when participants with a history of HF at baseline were excluded (eg, CV death/HF hospitalizations HR: 2.5; 95% CI: 1.1-5.7; *P* = 0.03; MACE HR: 2.6; 95% CI: 1.2-5.7; *P* = 0.02). However, with further adjustment for diabetes mellitus, HTN, CAC, and BMI, the association of continuous EMATc with MACE was no longer present (Table 6). In contrast, log NT-proBNP was associated with both outcomes in fully adjusted multivariable models (Supplemental Table 1). Heart rate was associated with both CV death/HF hospitalization and MACE in age, sex, race, and pro-BNP–adjusted models, but the association with MACE was no longer present in fully adjusted models.

**Table 6:** Association of EMAT, Heart Rate, and EMATc With Adverse Clinical Outcomes in Multivariable Analysis

	Number of Events^a^	EMATc		EMAT		Heart Rate		EMAT and Heart Rate			
		HR	*P* Value	HR	*P* Value	HR	*P* Value	EMAT HR	*P* Value	Heart Rate HR	*P* Value
CV death and heart failure hospitalization											
Base model	137	1.3 (1.2-1.4)	<0.001	1.3 (1.1-1.5)	<0.001	1.1 (1.03-1.3)	0.01	1.3 (1.1-1.5)	<0.001	1.2 (1.05-1.3)	0.003
Model 1	106	1.2 (1.1-1.4)	0.004	1.1 (0.9-1.3)	0.14	1.1 (1.02-1.3)	0.02	1.1 (0.98-1.3)	0.09	1.2 (1.03-1.3)	0.01
Model 2	73	1.3 (1.02-1.6)	0.03	1.2 (0.9-1.4)	0.20	1.2 (0.97-1.4)	0.10	1.2 (0.94-1.4)	0.17	1.2 (0.98-1.5)	0.08
MACE											
Base model	233	1.2 (1.1-1.4)	<0.001	1.2 (1.05-1.3)	0.004	1.2 (1.1-1.3)	<0.001	1.2 (1.1-1.3)	0.002	1.2 (1.1-1.3)	<0.001
Model 1	168	1.3 (1.1-1.4)	<0.001	1.1 (0.97-1.3)	0.12	1.2 (1.08-1.3)	<0.001	1.1 (0.99-1.3)	0.08	1.2 (1.1-1.3)	<0.001
Model 2	113	1.2 (1.04-1.5)	0.02	1.1 (0.9-1.3)	0.30	1.2 (1.03-1.4)	0.02	1.1 (0.9-1.3)	0.30	1.2 (1.04-1.4)	0.01
Model 3	91	1.2 (0.97-1.5)	0.09	1.0 (0.9-1.3)	0.60	1.1 (0.96-1.4)	0.13	1.1 (0.9-1.3)	0.50	1.2 (0.97-1.4)	0.10

CV = cardiovascular; other abbreviations as in Table 1.

The HR presented are based on 1 SD change of the exposure variable. SDs of EMAT, EMATc, and heart rate are shown in Tables 3 to 5.

Base model: Age, race, sex.

Model 1: Base model + NT-proBNP.

Model 2: Model 1 + troponin, QRS duration, eGFR.

Model 3: Model 2 + Diabetes, hypertension status, coronary artery calcium score, body mass index.

a Missing data led to a reduction in participants and events. Number of participants with missing data: NT-proBNP (n = 971); other covariates in Model 2 (another n = 737); for remainder of covariates in model 3 (another n = 120).

## Discussion

Acoustic cardiography is an emerging technology that may enhance remote monitoring of patients with HF.^1^^,^^3^^,^^26^ Herein we report the first demonstration of an association of EMAT and EMATc, key parameters measured by acoustic cardiography, with cardiac structure and function as assessed by CMRI, as well as clinical outcomes, in a large, multiracial community cohort.

The key findings of the current study were several-fold. 1) There was a broad range of EMAT values in the community with a nearly normal distribution. EMAT was associated with demographic characteristics, risk factors for and markers of cardiovascular disease, cardiac structure and function, and prognosis. 2) EMAT and EMATc were independently associated with lower LVEF in multivariable models that adjusted for many parameters including natriuretic peptide and Hs-cardiac troponin levels. Despite these associations, both an abnormal EMAT and EMATc had poor discrimination for a reduced LVEF. The discrimination for reduced LVEF of NT-proBNP was better than that for EMAT. 3) Although both higher EMAT and EMATc were associated with greater LV mass in fully adjusted models, they were associated with differential patterns of cardiac remodeling. Specifically, EMAT was associated with an increased LVEDV whereas EMATc was associated with markers of concentric hypertrophy (increased LV wall thickness and increased LV mass/EDV). The basis of this difference is likely explained by an association of heart rate, which is incorporated in the calculation of EMATc from EMAT, with smaller LVEDV and markers of LV concentric hypertrophy. 4) Both EMAT and EMATc were associated with an increased risk of adverse clinical outcomes in age-, race-, and sex-adjusted analyses. Notably, these associations of EMATc, but not EMAT, were independent of NT-proBNP levels. This difference is likely explained by the prognostic utility of the increased heart rate itself; and 5) The associations of NT-proBNP with CV death/HF or MACE were more robust than were those for either EMAT or EMATc.

Greater LV mass can be a result of either dilation of the LV chamber or thickening of the walls, and there is value in distinguishing between these 2 patterns of hypertrophy.^27^, ^28^, ^29^ Herein, we report the novel observation that EMAT was associated with ventricular dilation and EMATc with thick hypertrophy. The latter association is driven by the association of a higher heart rate with smaller LVEDV and increased LV mass/LVEDV, associations which have been demonstrated previously in the Multiethnic Study of Atherosclerosis.^30^ One prior smaller study with echo reported EMATc was significantly increased in those with eccentric rather than concentric hypertrophy.^16^ The basis for these differences between our conclusion and the prior study is unclear but their cohort consisted of those suspected of having HF, was a much smaller sample size, and was based on echocardiography rather than CMRI, the latter being the gold standard method to assess both cardiac structure and function.

Detection of reduced LVEF in the community could allow implementation of guideline-directed medical therapy which could improve outcome.^31^ A randomized clinical trial demonstrated the benefit of such an approach based on natriuretic peptide measurement in patients with known cardiovascular disease.^32^ In DHS-1, we previously demonstrated that the diagnostic utility of natriuretic peptide for detection of a reduced LVEF (≤55%) was suboptimal.^20^ Herein, we found that acoustic cardiography, although robustly associated with reduced LVEF in multivariable models (Table 3), also had suboptimal discriminatory power that was inferior to NT-proBNP alone. Furthermore, in the current study, neither EMAT or EMATc enhanced discrimination beyond NT-proBNP alone. In contrast, in a cohort of patients with signs and symptoms of decompensated HF or acute coronary syndrome, a panel of electrocardiographic and cardiac acoustic biomarkers measured by acoustic cardiography was more strongly associated with a low EF than BNP alone.^33^

EMAT and EMATc have been associated with adverse prognosis in patients with HF.^13^^,^^14^ In patients with heart failure with reduced ejection fraction discharged from an admission for decompensated HF, an algorithm incorporating EMAT, heart rate, and S_3_ intensity, as assessed by a wearable cardioverter defibrillator, detected HF decompensation well in advance of a subsequent HF readmission.^34^ One trial demonstrated improvement in clinical outcomes in patients discharged for acute decompensated HF with EMATc-guided, rather than usual, care.^35^ The current study was in a community cohort and thus inferences regarding the value of acoustic cardiography in patients with HF are limited. However, we demonstrated important differences between EMAT and EMATc, both in their associations with cardiac structure (LV dilation or thick hypertrophy^29^ as described previously), as well as in prognostic utility, and these differences can likely be ascribed to the heart rate itself. Heart rate was shown to be associated with the increased incidence of HF in the Multiethnic Study of Atherosclerosis.^30^ Overall, our data emphasize the need to tease out the contribution of heart rate when assessing the diagnostic or prognostic utility of EMATc, a concept that has not previously been described in the medical literature to our knowledge.

### Study Limitations

Acoustic cardiography was measured at baseline and thus these data should not be extrapolated to assess the prognostic utility of serial measurements of EMAT and EMATc over time, as by a wearable or implantable device. Specifically, longitudinal measurements, which permit detection of changes in these acoustic cardiographic parameters, may provide additional prognostic value beyond what was demonstrated herein with a baseline measurement and their utility as a screening tool for cardiomyopathy or worsening HF should be tested. Secondly, this study was conducted in a community cohort and thus its findings may not be generalizable to patients with an established diagnosis of chronic or acutely decompensated HF. The low prevalence of reduced LVEF may have impacted the estimate of the diagnostic utility of acoustic cardiography for its detection. Lastly, the modeling results were not corrected for multiple comparisons and should be interpreted with caution.

## Conclusions

In a community cohort, an increased EMAT was associated with a lower LVEF and adverse prognosis (Central Illustration). However, EMAT had poor discriminatory capacity for a reduced LVEF, which was inferior and not additive to NT-proBNP level. Furthermore, EMAT was not associated with subsequent adverse cardiovascular outcomes independently of NT-proBNP levels. Overall, these data indicate that measurement of NT-proBNP would be preferable to EMAT when screening a community cohort either to detect a reduced LVEF or to assess the subsequent risk of adverse cardiovascular outcomes.

**Central Illustration:**
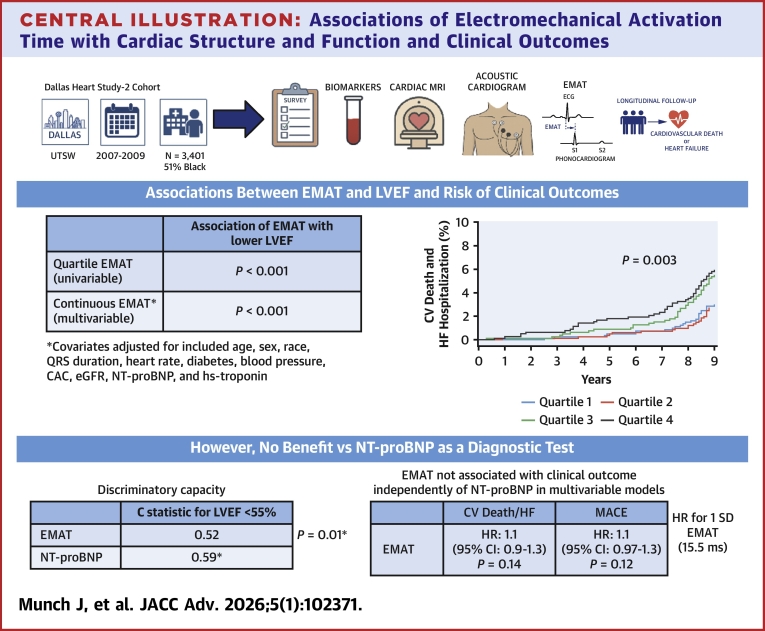
**Associations of Electromechanical Activation Time with Cardiac Structure and Function and Clinical Outcomes** In the Dallas Heart Study-2, a multiracial community cohort study, participants underwent acoustic cardiography which measured the electromechanical activation time (EMAT) as well as other detailed phenotyping including measurement of NT-proBNP levels and cardiac magnetic resonance imaging. Increased EMAT was associated with a lower left ventricular ejection fraction (LVEF) and risk of adverse cardiovascular outcomes including cardiovascular death and heart failure hospitalization. However, the discriminatory capacity for low LVEF (<55%) was suboptimal with a C-statistic of 0.52 and was inferior to NT-proBNP for these purposes. Furthermore, the associations of EMAT with adverse cardiovascular outcome were no longer present once NT-proBNP was incorporated into multivariable models, whereas NT-proBNP was associated with these outcomes in fully adjusted models. Together, these data demonstrate that EMAT was inferior to NT-proBNP measurement for screening a population to detect low LVEF or assess risk of adverse cardiovascular outcomes. Created in BioRender. Munch, J. (2025), https://BioRender.com/qnfqfoe. CAC = coronary artery calcium; CV death and HF hospitalization = cardiovascular death and heart failure hospitalization; eGFR = estimated glomerular filtration rate; HR = hazard ratio; hs-troponin = high-sensistivity-troponin; MACE = major adverse cardiac events; MRI = magnetic resonance imaging; NT-proBNP = N-terminal pro-B-type natriuretic peptide.

## Funding support and author disclosures

The DHS was supported by grants from the 10.13039/100000921Donald W. Reynolds Foundation and the 10.13039/100006108National Center for Advancing Translational Sciences (UL1TR001105). The Audicor system and sensors were provided by Inovise Medical (Oregon) to the Dallas Heart Study. Inovise had no role in planning the study, interpretation of data, or in preparation/review/approval/editing of the manuscript. Dr Mark Drazner was supported by the James M. Wooten Chair in Cardiology (University of Texas Southwestern Medical Center). Dr Pandey has received research support from the 10.13039/100000002National Institute of Health, 10.13039/100000968American Heart Association, Applied Therapeutics, 10.13039/100004337Roche, Ultromics, 10.13039/100005564Gilead Sciences, and Astra Zeneca and has received honoraria outside of the present study as an advisor/consultant for Tricog Health Inc, Lilly USA, Rivus, Cytokinetics, Roche Diagnostics, Axon therapies, Medtronic, Edward Lifesciences, Science37, Novo Nordisk, Bayer, Medical AI, Baylor Scott and White Research Institute, Tenax, Boehringer Ingelheim, Tourmaline Bio, Merck, Sarfez Pharmaceuticals, Emmi Solutions, Semler Scientific, Ultromics, Merck, Encarda, Kieele Health, Anumana, and Acorai. Dr Grodin reports personal fees from Pfizer, Alnylam, Eidos/BridgeBio, AstraZeneca, Alexion, Lumanity, Novo Nordisk, Ultromics, Intellia, and Tenax Therapeutics and grant support from 10.13039/100000050NHLBI (R01HL160892), 10.13039/100004319Pfizer (67656485), Eidos/BridgeBio, and Texas Health Resources Clinical Scholars Fund. Mr Ayers reports NIH statistical consulting fees. All other authors have reported that they have no relationships relevant to the contents of this paper to disclose.
