# Supplementary material for: Associations of Electromechanical Activation Time With Cardiac Structure and Function and Clinical Outcomes
Source: JACC Adv. 2025 Nov 27;5(1):102371. doi: 10.1016/j.jacadv.2025.102371 (PMC12702212; doi:10.1016/j.jacadv.2025.102371)
Supplement: Supplemental Table and Supplemental Figures [file mmc1.docx]

**Supplemental Table 1. Association of log NT-proBNP with clinical outcomes**

|  | **Hazard ratio** | **P** |
| --- | --- | --- |
| **CV death/HF hospitalization** | | |
| Base model | 2 (1.8 – 2.4) | <0.001 |
| Model 1 | 1.6 (1.3 – 2) | <0.001 |
| Model 2 | 1.6 (1.2 – 2.1) | <0.001 |
| **MACE** | | |
| Base model | 1.7 (1.5 – 2.0) | <0.001 |
| Model 1 | 1.5 (1.2 – 1.8) | <0.001 |
| Model 2 | 1.5 (1.2 – 1.9) | <0.001 |

NT-proBNP was log transformed in all models.

Base model: Age, race, sex

Model 1: Base + troponin, QRS duration, eGFR, EMAT, heart rate

Model 2: Model 1 + Diabetes, Hypertension status, coronary artery calcium score, body mass index

**Supplemental Figure 1A. Heart rate and CV death/HF hospitalization**

P=0.04

CV death and

HF hospitalization (%)

**Supplemental Figure 1B. Heart rate and MACE**

MACE (%)

P=0.006
